# Molecular Mechanisms Underlying Hepatocellular Carcinoma Induction by Aberrant NRF2 Activation-Mediated Transcription Networks: Interaction of NRF2-KEAP1 Controls the Fate of Hepatocarcinogenesis

**DOI:** 10.3390/ijms21155378

**Published:** 2020-07-29

**Authors:** Effi Haque, M. Rezaul Karim, Aamir Salam Teeli, Magdalena Śmiech, Paweł Leszczynski, Dawid Winiarczyk, Emil D. Parvanov, Atanas G. Atanasov, Hiroaki Taniguchi

**Affiliations:** 1 Institute of Genetics and Animal Biotechnology of the Polish Academy of Sciences, 05-552 Jastrzębiec, Poland; e.haque@ighz.pl (E.H.); teeliaamir7@gmail.com (A.S.T.); m.smiech@ighz.pl (M.Ś.); p.leszczynski@ighz.pl (P.L.); d.winiarczyk@ighz.pl (D.W.); atanas.atanasov@univie.ac.at (A.G.A.); 2 Department of Biotechnology and Genetic Engineering, Jahangirnagar University, Savar, Dhaka 1342, Bangladesh; rkarimcu@bgeju.edu.bd; 3 Division BIOCEV, Institute of Molecular Genetics, Academy of Sciences of the Czech Republic, 420 Prague, Czech Republic; eparvanov@gmail.com; 4 Ludwig Boltzmann Institute for Digital Health and Patient Safety, Medical University of Vienna, 1090 Vienna, Austria; 5 Institute of Neurobiology, Bulgarian Academy of Sciences, 1113 Sofia, Bulgaria; 6 Department of Pharmacognosy, University of Vienna, 1090 Vienna, Austria

**Keywords:** NF-E2-related factor 2, transcription factor, redox homeostasis, oxidative stress, hepatocellular carcinoma

## Abstract

NF-E2-related factor 2 (NRF2) is a basic leucine zipper transcription factor, a master regulator of redox homeostasis regulating a variety of genes for antioxidant and detoxification enzymes. NRF2 was, therefore, initially thought to protect the liver from oxidative stress. Recent studies, however, have revealed that mutations in NRF2 cause aberrant accumulation of NRF2 in the nucleus and exert the upregulation of NRF2 target genes. Moreover, among all molecular changes in hepatocellular carcinoma (HCC), NRF2 activation has been revealed as a more prominent pathway contributing to the progression of precancerous lesions to malignancy. Nevertheless, how its activation leads to poor prognosis in HCC patients remains unclear. In this review, we provide an overview of how aberrant activation of NRF2 triggers HCC development. We also summarize the emerging roles of other NRF family members in liver cancer development.

## 1. Introduction

Liver cancer is one of the most troublesome human malignancies, with an annual incidence of around 600,000 worldwide (https://doi.org/10.3322/canjclin.55.2.74). Among different types of liver cancer, hepatocellular carcinoma (HCC) is the sixth most common malignancy of liver (http://gco.iarc.fr/) and the eighth leading cause of cancer-related deaths in Europe (http://gco.iarc.fr/today). Chronic hepatitis B (HBV) or C (HCV) virus infection, alcohol intake, diabetes, fatty liver disease, and chronic liver injury cause permanent hepatocellular damage, hepatocyte regeneration, and inflammation, which are key risk factors for HCC. Infection with HBV or HCV and alcohol-induced hepatocarcinogenesis are associated with oxidative stress in the liver [1,2,3,4,5]. Oxidative stress also contributes to genomic instability, and the altered gene expression leads to HCC development [6,7,8]. Moreover, oxidative stress has been suggested to cause cancer-specific gene mutations in the cell cycle, apoptosis, and various processes of the regeneration cycle, which may lead to liver damage [9,10,11,12].

NF-E2-related factor 2 (NRF2) transcription factor is activated by oxidative stress, and recent studies have suggested that the aberrant activation of NRF2 triggers hepatomegaly and HCC development [13,14], however, this factor also acts to protect the liver from oxidative stress. The protein interaction of NRF2 and Kelch-like ECH-associated protein 1 (KEAP1) is known to orchestrate the NRF2-dependent oxidative stress response to maintain liver homeostasis. In the presence of oxidative stress, KEAP1 is degraded in the cytoplasm, and due to the degradation, NRF2 is released from KEAP1 [15,16]. Thereafter, NRF2 is phosphorylated and translocates into the nucleus, and induces a series of cytoprotective genes by binding to the antioxidant response element (ARE) after heterodimerization with small musculoaponeurotic fibrosarcoma oncogene homolog (Maf) proteins [17,18,19]. More importantly, several studies have demonstrated that somatic mutations occur in the coding region of NRF2 and are associated with poor prognoses and overall low survival rates in several cancers [20,21,22,23]. The mutations are mostly located in the DLG or ETGE motifs of NRF2 and have been reported to impair the NRF2 binding ability to KEAP1, which in turn leads to aberrant nuclear accumulation of NRF2 [21,23,24]. In this review, we describe how the aberrant transcriptional activation of NRF2 caused by its nuclear accumulation may develop HCC at molecular level.

## 2. Oxidative Stress-Dependent HCC Pathogenesis

Multiple genetic and epigenetic changes are involved in the development of HCC. Many studies have revealed that oxidative stress is one of the causes linked to tumor initiation and progression by disrupting the normal cellular redox homeostasis [25,26,27]. Oxidative stress also induces mitochondrial dysfunction, accelerates telomere shortening, causes DNA damage, and is associated with mutations of apoptosis-specific genes in HCC [28,29,30,31,32]. An increased level of reactive oxygen species (ROS), generated by Kupffer cells during hepatic inflammation, has been associated with the progression of liver pathologies [33]. Similarly, oxidative stress disrupts intracellular signaling pathways and contributes to HCC. Alterations in nuclear factor-kappa beta (NF-κB), peroxisome proliferator-activated receptor alpha (PPARα), mitogen-activated protein kinase (MAPK), extracellular signal-regulated kinases (ERK), and transforming growth factor β1 (TGF-β1) pathways due to oxidative stress are commonly associated with HCC, as all these pathways are involved in the activation of cellular proliferation and survival [34,35,36,37,38,39]. Moreover, HCV infection enhances NF-κB and TGF-β1 expression through the production of ROS and activation of p38-MAPK, ERK, and JNK, and thus promotes the development of hepatic fibrosis [35,37]. Rodent models of DEN/CCl4-induced hepatocarcinogenesis display a significant upregulation of liver-specific NF-κB and TGF-β1/Smad3 signaling [40]. In contrast, HepG2 HCC cells exposed to cadmium telluride quantum dots-induced oxidative stress exhibit decreased levels of Glutathione-SH and Bcl2 and increased expression of NRF2 together with apoptosis induction due to the activation of the MAPK-JNK pathway [36]. These studies suggest that multiple intracellular signaling pathways are activated by oxidative stress, and notably, either carcinogenic or anticarcinogenic pathways are triggered in a context-dependent manner.

## 3. Dysregulation of NRF2-KEAP1 Physical Interaction Triggers Several Types of Cancer

Several studies have indicated that the NRF2-KEAP1 signaling pathway functions as an oxidative stress sensor [16]. KEAP1 is an adapter protein for the E3 ubiquitin ligase complex that controls the stability and accumulation of NRF2 [41]. Under normal conditions, KEAP1 binds to NRF2 and directs it to Cullin 3-RING E3 (CUL3 E3) ligase for ubiquitination and subsequent proteasomal degradation [42,43]. Upon exposure to oxidative or electrophilic stresses, KEAP1-mediated proteasomal degradation of NRF2 is inhibited and leads to NRF2-mediated transcription of various genes in several types of tissue, including liver. NRF2 is a member of the Cap’n’Collar (CNC) subfamily of basic leucine zipper (bZIP) transcription factors that regulate a wide variety of genes for antioxidant and detoxification enzymes [44,45]. This activity relies on its transactivation capacity and its heterodimeric partner, small MAF transcription factor [46]. The NRF2 protein consists of seven conserved NRF2-ECH homology (Neh) domains. Neh1 contains CNC-bZIP domain via which NRF2 dimerizes with Maf [46]. The consensus binding site of NRF2 is the ARE sequence (TGACNNNGC) [47,48,49]. The N-terminal region contains the highly conserved Neh2 domain, which negatively regulates the transcriptional activity of NRF2. The Neh2 domain of NRF2 contains DLG and ETGE motifs, which are the binding sites for KEAP1 [50,51,52]. Alternatively, repression of Nrf2 is achieved by interactions of Neh7 with the DNA-binding domain of retinoic X receptor α [53]. Neh6 is target for E3 ubiquitin ligase β-TrCP leading to degradation [54]. The Neh3–5 domains are thought to bind to transcriptional system [55,56]. KEAP1 is a member of BTB-Kelch family of proteins. The BTB domain is N-terminally situated and is responsible for homodimerization of KEAP1 and interaction with CUL3 [57]. The C-terminal Kelch domain binds to the ETGE motif or DLG motif of NRF2 [51]. The Kelch domain forms a six-bladed β-propeller structure, and this domain is evolutionarily conserved among species. Moreover, among the six Kelch blades, four β-strands are conserved in each blade [58]. Recent studies have provided insight into how mutations disturb the structure of the BTB-Kelch domain that is responsible for NRF2 binding. It has been reported that one single-point mutation, a proline substitution for serine 383 (S383P) in KEAP1, significantly reduces the ability of the Kelch domain to bind with the Neh2 domain of NRF2 [58]. Moreover, KEAP1 substitution mutations of cysteine residues Cys273S/A and Cys288S/A do not affect the ability of KEAP1 to interact with NRF2, but they impair the KEAP1-mediated degradation of NRF2 [59,60]. Notably, many studies have revealed that the loss of interaction between NRF2 and KEAP1 causes tumor development in multiple cancer types. Furthermore, it has been reported that the deletion of Exon2 in NRF2, which reduces interaction with KEAP1, causes tumor development in lung and liver cancer [61]. Together, a tightly regulated balance of NRF2 and KEAP1 interaction is essential to protect cells or tissues from oxidative stress, and the failure of that mechanism (e.g., mutations of critical amino acids) triggers cancer development.

## 4. Mutations in NRF2 and KEAP1 Functional Domains Induce HCC Development

**Figure 1:**
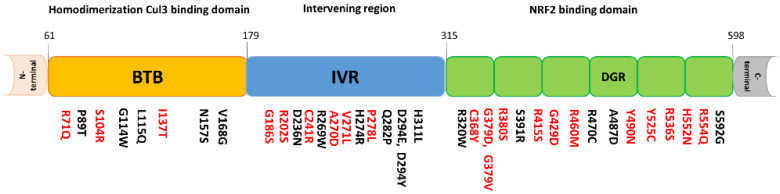
Human Kelch-like ECH-associated protein 1 (KEAP1) mutations (liver cancer) identified by the International Cancer Genome Consortium. The amino acid positions of the identified mutations of KEAP1 are shown and the amino acid positions in red color indicate the location of mutations that are conserved among several species (Human, Mouse, Bovine, and Zebrafish). The ^61^BTB^179^ domain is required for homodimerization of Keap1 by interactions with the Cul3 based E3 ubiquitin ligase system. The ^315^DGR^598^ or 6 Kelch-repeat domain binds to NRF2 through Neh2 domain of NRF2. ^180^IVR^314^ domain between BTB and DGR domain important sensing oxidative stress and xenobiotic stimuli via modification of its four cysteine residues by electrophiles.

The International Cancer Genome Consortium (ICGC; https://icgc.org/) has identified somatic mutations in the NRF2 gene of human lung, liver, breast, head, and neck cancer patients. Notably, these mutations were mostly located within the DLG and ETGE motifs, which provides NRF2 with gain-of-function activity in different cancer types [21,23,62]. Therefore, we surveyed the ICGC database and compiled the HCC somatic mutations found in DLG and ETGE motifs (Table 1). KEAP1 mutations are also found within the BTB, IVR, and Kelch domains (Figure 1). Comprehensive genomic analyses have identified somatic mutations in the *NRF2* and *KEAP1* genes in various types of cancer [21,23,63]. Interestingly, mutations in *KEAP1* and *NRF2* are mutually exclusive and rarely occurred in the same cancer cell [64]. Whole-exome sequencing has identified 6.4% of the somatic mutations in *NRF2* in HCC patients [24]. Somatic *NRF2* and *KEAP1* mutations were most often found in lung squamous cell carcinoma, esophageal carcinoma, uterine corpus endometrial carcinoma, lung adenocarcinoma, head and neck cancers, and HCC; the overlapping somatic *NRF2* or *KEAP1* mutations are associated with a sustained NRF2 activation phenotype [21,23]. In 995 lung cancer cases, 423 cases were estimated to have constitutive NRF2 activation, and 165 cases harbored either *NRF2* or *KEAP1* mutations with higher expression of NRF2 target genes, including Aldo-Keto Reductase Family 1 Member B10 (*AKR1B10*), Aldo-Keto Reductase Family 1 Member B15 (*AKR1B15*), Glutathione Peroxidase 2 (*GPX2*), Thioredoxin Reductase 1 (*TXNRD*1), Glutamate-Cysteine Ligase Modifier Subunit (*GCLM*), and Glutamate-Cysteine Ligase Catalytic Subunit Glutamate-Cysteine Ligase Catalytic Subunit (*GCLC*) [21]. It has been reported that NRF2 gain-of-function mutations are one of the possible triggers of HCC [65,66]. In an experimental rat model of hepatocarcinogenesis, it was found that the *NRF2* gene was frequently mutated or activated during the early stage of the tumorigenic process [66]. This suggests that NRF2 plays a potent role for the initiation of HCC and is mandatory for the development of preneoplastic lesions. The role of constitutive NRF2 activation is well established in chemo- and radio resistance in various tumors [67,68]. In Lung Squamous Cell Carcinoma (LSCC) mice models developed by *KEAP1* deletion, the persistent activation of NRF2 has been suggested to contribute to increased tumor formation, metastasis, and resistance to oxidative stress and irradiation [67]. The NRF2 DLG and ETGE motifs have been reported as driver mutations in several types of cancer including HCC [62,65,66]. A recent CRISPR/Cas9 genome-wide screening study demonstrated that KEAP1 depletion causes aberrant NRF2 transcriptional activity as well as high chemoresistance. Upregulation in NRF2-target gene expression including NAD(P)H-quinone oxidoreductase 1 (*NQO1*), *GPX2,* and *TXNRD1* was also observed [69]. Moreover, microdeletion of *NRF2* exon 2 (where the DLG and ETGE domains are located) in JHH cells is associated with increased NRF2 target gene expression and is similar to HCC cell lines containing *KEAP1* mutations [61]. NRF2 DLG and ETGE mutations that demonstrate a loss of interaction with KEAP1, are localized to the nucleus and exert sustained target gene activation. In fact, *NRF2* mutations occur frequently in the DLG or ETGE motifs and activate Nqo1, Gclc, and Gsta4 pathways. This suggests that *NRF2* mutations are able to enhance NRF2 transcriptional activity [62]. Mutation in the DLG motif of NRF2 induces ARE-regulated PPP enzyme transcription, which is required for cell growth and proliferation [65,70]. NRF2 also translocates into the nucleus in HCV-infected hepatocytes and causes mouse double minute 2 homolog (MDM2)-mediated retinoblastoma protein (Rb) degradation. This subsequently induces HCC progression [71]. Overall, these findings suggest that either the *NRF2* DLG/ETGE mutations or KEAP1 mutations induce aberrant NRF2 activity and may induce HCC through NRF2-ARE pathway activation. Moreover, methylation of the *KEAP1* promoter has been reported to induce cancer development and chemo- and radio- resistance in multiple cancer types [72,73]. It has been determined that the most frequent somatic mutations found in HCC are telomerase reverse transcriptase (*TERT*) promoter mutations, identified in premalignant lesions in cirrhosis [74]. TERT is essential for telomere elongation and maintenance during cell division. *TERT* promoter mutations are associated with increased telomerase activity, which displays enhanced *NRF2* expression and inhibited glycogen accumulation [75]. As such, it appears there are many possible pathways to trigger liver cancer via aberrant NRF2 transcriptional activity, and further phenotypic validation of the roles of these pathways in liver cancer development merits investigation.

## 5. Aberrantly Activated NRF2 Targets Several Gene Expressions in HCC

As summarized in Table 2, it has been suggested that excessive NRF2 transcriptional activity promotes the development of liver cancer by regulating the expression of various genes. During oxidative stress, cells initiate an adaptive response that upregulates expression of a large array of cytoprotective genes. The battery of genes is regulated through NRF2 binding to the ARE consensus binding sequence, which includes glutamate-cysteine ligase (*GCL*), thioredoxin reductase 1, NAD(P)H-quinone oxidoreductase 1 (*NQO1*), and heme oxygenase-1 (*HO-1)* [76]. In healthy cells, HO-1 exhibits its cytoprotective effect through detoxification and ROS scavenging mechanisms that decrease the possibility of tumor initiation. However, in a tumor microenvironment, sustained NRF2 expression persists due to stress, which tightly regulates the expression of HO-1. Thus, in growing tumors, HO-1 plays a pro-tumorigenic role by increasing tumor cell proliferation and metastasis and blocking cell death [77,78].

The extracellular matrix metalloproteinase MMP-9 demonstrates a crucial role in HCC invasion and progression [79]. Many studies have suggested that MMP-9 is a prognostic biomarker to predict tumor invasiveness and recurrence in HCC patients [80,81]. Moreover, it was found that 98 samples displayed MMP-9 positive expression in 143 HCC tissue samples. It has been reported that the *MMP-9* gene expression has a strong correlation with the metastatic potential of HCC cell lines (MHCC97-L, MHCC97-H, and HCCLM6) and its expression was significantly increased in an in vitro HCC invasion model [79]. This evidence suggests that MMP-9 plays a critical role in HCC invasiveness and metastasis. A positive correlation between *NRF2* and *MMP-9* expression in human HCC samples and HCC cell lines has been reported [82]. Moreover, NRF2-mediated induction of MMP-9 plays an important role in cell proliferation and invasion [82].

The presence of a strong correlation between NRF2 and the PI3K-Akt signaling pathway has been demonstrated in driving metabolic gene expression and increased cellular proliferation [83]. In many cancers, the PI3K-Akt pathway is constitutively active and may be responsible for increasing the nuclear levels of NRF2 by inhibiting the GSK3-mediated degradation of NRF2.

Peroxiredoxin 1 (*PRDX1*), a NRF2 target gene, is known to act as an oxidative stress sensor and promote liver cancer growth [84,85]. In HepG2 cells, a significant decrease in cell proliferation and upregulation of proapoptotic genes BAX and Caspase 3 were observed when *PRDX1* was silenced, suggesting the prosurvival and tumorigenic roles of *PRDX1*. Furthermore, proteomic analysis has revealed changes in expression and oxidation of proteins involved in central metabolism and tumor growth, indicating that PRDX1 is one of metabolic reprogramming factors in cancer cells [86].

Methylenetetrahydrofolate dehydrogenase 1–like (MTHFD1L) is an enzyme, involved in the folate cycle which is also known as a target gene of NRF2. Transcriptome sequencing of HCC patients and The Cancer Genome Atlas (TCGA) data showed that MTHFD1L is significantly overexpressed in different cancers, including HCC. During HCC cell proliferation, the folate cycle provides nutrition to the cells by supplying metabolites for NADPH and DNA synthesis. The *MTHFD1L* promoter has three ARE sequence elements and is transcriptionally controlled by NRF2. Genetic knockdown (KD) of either *NRF2* or *MTHFD1L* or inhibition of the folate cycle through antifolate drug can inhibit liver cancer cell proliferation by increasing oxidative stress, altering the metabolic program, and sensitizing HCC cells to sorafenib treatment [87].

In cancer cells, the aberrant activation of NRF2 helps their metabolic adaptations through regulating the key genes involved in glucose metabolism pathways. It has been reported that the effect of NRF2 on the regulation of glucose metabolism is partly through suppressing the transcription of miR-1 and miR-206. The miR-1 and miR-206 regulate their target genes of the PPP (pentose phosphate pathway) (Glucose-6-phosphate dehydrogenase *(G6PD)*, Phosphogluconate Dehydrogenase *(PGD)*, Transketolase *(TKT)*, and Transaldolase 1 *(TALDO1)*) [88], suggesting that suppression of these miRNAs expression caused by NRF2 aberrant activation may enhance tumor development through glucose metabolism deficiency. Additionally, suppressed expression of miR-1 also has been proposed to be important in liver cancer cell growth [89]. On the other hand, in a resistant-hepatocyte rat model of HCC, microRNA profiling revealed an upregulation of miR-200a at the very early stage of tumorigenesis. miR-200a downregulates KEAP1 in several cancer types including HCC [90,91] and it promotes induced expression of *NQO*-1 and *GSTA*-4, and *GCLC* genes which are the direct target of NRF2 [91]. These findings suggest that NRF2 plays important roles in the promotion of liver cancer cell growth through the regulation of several genes and microRNAs.

## 6. Aberrant Activation of NRF2, a Critical Regulator of Lipid and Cholesterol Metabolism, Leads to HCC

Non-alcoholic fatty liver disease (NAFLD) is considered one of the risk factors of HCC. NAFLD includes a variety of liver pathologies including the accumulation of triglycerides in the hepatocytes, liver inflammation, and non-alcoholic steatohepatitis (NASH) that leads to cirrhosis and thereafter HCC [103,104,105]. The most important mechanism of NASH pathogenesis is increased hepatic iron accumulation, as well as oxidative DNA damage [106]. Highly proliferative cancer cells show strong affinity towards lipid and cholesterol metabolisms [107], and high levels of cholesterols and lipids are now considered hallmarks of many aggressive cancers [108,109,110,111,112]. It is also evident that hepatic lipid and fatty acid overload are related to the development of HCC [113]. The pathogenic role of NRF2 for the initiation and development of hepatic steatosis was described previously [114]. Additionally, enhanced NRF2 activity augments hepatic steatosis and increased lipid deposition in the liver has been reported. In leptin-deficient mice, constitutive activation of NRF2 via KEAP1-KD established insulin resistance, inhibited the accumulation of lipids in adipose tissue, and subsequently increased hepatic steatosis. [114]. Moreover, dysfunction in the starvation-induced hepatic lipid droplets (LDs) synthesis in liver-specific Atg5 (L-Atg5)-deficient mouse livers was associated with sustained NRF2 activation [115]. Though it has been reported that NRF2 activation may reduce cholesterol injury by regulating the lipid homeostasis, how the hyperactivation of this transcription factor in NAFLD leads to HCC needs to be further clarified.

NAFLD results from unbalanced lipid metabolism. Forkhead box protein A1 (FOXA1) is a triglyceride synthesis inhibitor, and it is well known to lower fatty acid uptake [116]. Thus, FOXA1 is thought to be an antisteatotic regulator in lipid metabolic pathways hepatocytes. Notably, it was found that excessive cholesterol synthesis causes the accumulation of NRF2. The accumulated NRF2 suppresses the expression of FOXA1 [117], and the downregulation of FOXA1 has been found in human and rat NAFLD [116]. In this regard, the disruption of lipid metabolism and oxidative stress have been reported as the main causes of NAFLD, and NRF2 is related to lipid homeostasis [118,119]. Peroxisome proliferator-activated receptor gamma (PPARγ) is also one of the most studied lipid metabolism regulators in hepatocytes and it contributes to the development of NAFLD [120]. Interestingly, it has been reported that *PPARγ* gene expression is regulated by NRF2 [121]. Moreover, it was found that liver-specific *Nrf2*-KO mice with high-fat diet (HFD) had less steatosis and inflammation with less hepatic triglyceride levels and decreased PPARγ activity [100]. Furthermore, constitutively activated NRF2 signaling in *Keap1*-KD mice fed a HFD exhibited greater lipogenic gene expression, inflammation, and increased hepatic steatosis [122]. These findings suggest that aberrant activation of NRF2 helps to trigger development of NASH or NAFLD, therefore, gain-of-function type mutations in NRF2 may initiate the development of HCC by inducing NASH or NAFLD. Nevertheless, since the NRF2-controlled gene network contributing to the promotion of HCC is not clear, further studies are needed to investigate how NRF2 induces NASH or NAFLD at the molecular level.

## 7. Emerging Mechanism of NRF2 Activation-Induced HCC

ROS is usually considered to be carcinogenic and several chemopreventive strategies for the usage of NRF2 have been proposed [123,124]. Since NRF2 is widely known to be a potent protector in anti-oxidative response, a question arises as to how NRF2 DLG and ETGE mutations lead to increased malignancy of HCC and trigger its resistance to chemotherapy. This issue has been discussed very intensively in several reviews [14,125] and an excellent hypothesis is proposed by Sporn and his group that the role of NRF2 can be altered depending on the stage of tumor progression [126]. They proposed a model in which enhancement of NRF2 activity can protect advanced tumors from the cytotoxic effects of ROS that are induced by oncogenic signaling whereas NRF2 activation acts as protective for tumor establishment in normal condition. Interestingly, oncogenic gene mutations such as K-RasG12D, B-RafV619E enhanced transcription of NRF2 with elevated NRF2 target gene expression and lowered intracellular ROS [127]. Therefore, it is assumed that aberrant transcriptional activity induced by high expression or mutation of NRF2 may lead to malignancy in combination with other factors (for example, mutation of oncogene leads cells to early cancer state or abnormality of metabolic state changes in cellular environment). In these conditions, the cells can be shifted to malignancy when they are induced to become HCC progenitor cells. Interestingly, it has been shown that elevated p62 levels aberrantly activate NRF2 transcriptional activity, which induces HCC pathogenesis by accelerating the survival of HCC-initiating cells [128]. p62, which is encoded by Sequestosome-1 (SQSTM1), is an autophagy adaptor. It activates NRF2 through inactivation of Keap1 [129]. Autophagy is an evolutionary conserved cellular mechanism that maintains cell homeostasis by targeting damaged organelles or mistranslated proteins for lysosomal degradation. Atg7 deletion mice develop hepatocellular adenoma accompanied by aberrant accumulation of p62 followed by NRF2 activation [130]. The study further elucidated the role of p62 in aberrant activation of NRF2 in HCC. The persistent activation of NRF2 is associated with p62 accumulation and the development of HCC in vitro [130]. Furthermore, high levels of p62 expression activates NRF2 and mTORC1 in HCC [128]. Consequently, this NRF2 activation spares HCC-initiating cells from oxidative stress-induced cell death [128]. This is supported by the fact that the kinase-dead mutation of p62 (S349A) in Human hepatoma cell line-1 (Huh-1) cells significantly reduces colony formation capacity with decreased *NQO1* mRNA expression [131]. Moreover, a xenograft experiment using a nude mouse demonstrated that the tumor formation capacity of mutant Huh-1 cells (p62 KO and S349A) is reduced as compared to the wild-type. Additionally, p62-mediated NRF2 activation in HCC cells facilitates the glucuronate pathway and glutathione synthesis in HCV positive HCC [132]. NRF2 activation contributes to metabolic reprogramming in HCC harboring phosphorylated p62 [133]. This leads to increased cell proliferation and increases tolerance to anti-cancer drugs in HCC [132]. These findings clearly demonstrate that sustained activation of NRF2 by p62 activation is responsible for HCC pathology, suggesting that NRF2 and KEAP1 mutations, as well as aberrant p62 activation enhance the growth of HCC cells through metabolic dysregulation. NRF2 DLG and ETGE mutations lose the capacity to interact with KEAP1 and localize mainly to the nucleus. Accordingly, these mutations may activate NRF2 target gene expression and exhibit a similar phenotype to p62 activation in the liver. Future studies on the effect of NRF2 DLG and ETGE mutations in combination with autophagy and/or other cellular function in HCC are warranted.

## 8. Emerging Roles of the CNC Family of Transcription Factors in HCC

The CNC bZIP family of transcription factors, which comprises four closely related factors, NRF1, NRF2, NRF3, and p45 NF-E2, have developmental and homeostatic functions [134,135]. The CNC gene encodes different proteins, with evolutionary conservation between *Drosophila* CNC isoform and mammalian NRF1, NRF2, and NRF3 [136]. With similar binding and expression profiles, NRF1, NRF2, and NRF3 reside outside of the nucleus under normal conditions [137]. NRF2 resides in the cytoplasm and NRF1 and NRF3 in the endoplasmic reticulum [137,138]. All three transcription factors are essential for maintaining redox homeostasis and directing cellular stress responses. Much like NRF2, NRF1 contains the Neh2 domain and NRF3 does not [139]. NRF-encompassing amino acids 171–244 of Neh2 share 72% homology with the Neh2 domain of NRF2 [137]. In addition, NRF1 has conserved DLG and ETGE motifs within the Neh2 domain, which are essential for KEAP1–NRF1 interaction [140,141]. Moreover, a study indicated that NRF1 expression was significantly reduced in KEAP1-KO H1299 cells, suggesting that KEAP1 stabilizes NRF1 [142]. On the other hand, it has been reported that cytoplasmic localization of NRF1 is independent of KEAP1 whereas KEAP1 physically interacts with NRF1 [137]. Therefore, it is still not clear whether KEAP1 regulates NRF1 function and further study is needed. NRF1 and NRF2 have overlapping targets binding ARE-containing genes, but have distinctive roles [143]. It has also been reported that NRF1 and NRF2 simultaneously control the basal expression of ARE-containing genes in fibroblasts [144]. Likewise, NRF1-3 are known to regulate proteasome gene expression [145,146,147]. Moreover, the role of NRF1 and NRF3 has been indicated in human cancers, including HCC [148,149,150]. These results suggest that NRF1 and NRF3 mutations and aberrant gene expressions may trigger HCC. The molecular regulation and biological function of NRF3 in cancer cells have been elucidated [150,151,152]. When exposed to stress, NRF3 translocates to the nucleus, heterodimerizes with the small Maf proteins similarly to NRF2 via ARE, and activates U2AF homology motif kinase 1 (*UHMK1*) gene expression [150]. This study suggests that NRF3 functions as an inducible transcription factor in cancer progression. Moreover, analysis of TCGA data revealed that NRF3 was highly expressed in HCC tissues, and its expression was positively correlated with tumor grade and stage [149]. In addition, NRF3 deficiency has been revealed to predispose to T-cell lymphoblastic lymphoma when exposed to carcinogens [153]. Likewise, liver-specific inactivation of the *Nrf1* gene in adult mice has been reported to trigger NASH [154], suggesting that the proper activity of NRF1 and NRF3 blocks carcinogenesis, including liver cancer. Therefore, the roles of NRF1 and NRF3 and their mutational effects in HCC merit investigation.

## 9. Conclusions

**Figure 2:**
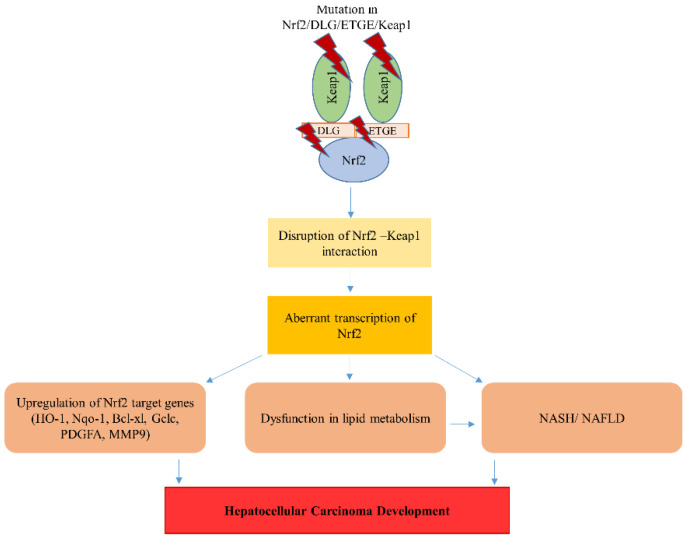
Mutations in NRF2 or Keap1 cause aberrant accumulation of NRF2 in the nucleus that leads to an increase in NRF2 target genes. This aberrant activation of NRF2 dysregulates the lipid metabolism responsible for the non-alcoholic steatohepatitis (NASH)/non-alcoholic fatty liver disease (NAFLD) pathology. Consequently, these events lead to the development of HCC.

In this review, we discussed the current evidence on how aberrant NRF2 transcriptional activity causes HCC development (Figure 2). Moreover, we reviewed the mutations found in ICGC databases in the specific domain that is essential for the KEAP1-NRF2 interaction impact on HCC development. The aberrant activation of NRF2, the dark side of this protein expression, induces the transcription of a series of cytoprotective and xenobiotic-metabolizing genes. Furthermore, align with NRF2, other CNC family members, NRF1 and NRF3, are also dysregulated during HCC development. Hepatocytes are in a continuous struggle to maintain cellular homeostasis, owing to diverse physiological functions of the liver. In a diseased microenvironment, when NRF2 over-activation is induced through mutation, epigenetic changes, competition or other constitutive changes, and cellular responses are variable. Thus, how the hyperactivity of NRF2 leads to drug resistance and tumor development is the hotspot of future research. Further studies are needed to clarify the underlying mechanisms and investigate the role of NRF2 mutations in the development of liver cancer.

## Figures and Tables

**Table 1:** NF-E2-related factor 2 (NRF2) mutations found in NRF2-ECH homology 2 (Neh2) domain ETGE and DLG motif of hepatocellular carcinoma (HCC) patients (International Cancer Genome Consortium (ICGC) database).

Mutation ID	Genomic DNA Change	Type	Motif	Substitution to	Clinical Significance	Occurrence of Mutation
MU871836	chr2:g.178098959T > C	single base substitution	DLG	G	Likely pathogenic	5
MU866686	chr2:g.178098953C > T	single base substitution	DLG	E	ND	2
MU1327674	chr2:g.178098960C > T	single base substitution	DLG	N	Likely pathogenic	2
MU29615597	chr2:g.178098959T > G	single base substitution	DLG	A	ND	1
MU83818151	chr2:g.178098954C > T	single base substitution	DLG	R	Pathogenic/Likely pathogenic	1
MU1324215	chr2:g.178098960C > G	single base substitution	DLG	H	Likely pathogenic	1
MU830878	chr2:g.178098956A > C	single base substitution	DLG	R	ND	3
MU1330977	chr2:g.178098957G > A	single base substitution	DLG	F	ND	1
MU825005	chr2:g.178098800T > C	single base substitution	ETGE	G	ND	4
MU7421282	chr2:g.178098809T > C	single base substitution	ETGE	G	ND	2
MU29777568	chr2:g.178098806G > A	single base substitution	ETGE	I	ND	2
MU29708787	chr2:g.178098799T > G	single base substitution	ETGE	D	ND	2
MU1890585	chr2:g.178098804C > A	single base substitution	ETGE	C	ND	2
MU1332094	chr2:g.178098804C > T	single base substitution	ETGE	S	ND	2
MU3162037	chr2:g.178098809T > A	single base substitution	ETGE	V	Likely pathogenic	2
MU128988244	chr2:g.178098803C > G	single base substitution	ETGE	A	ND	1
MU1804262	chr2:g.178098799T > A	single base substitution	ETGE	D	ND	1
MU41238347	chr2:g.178098804C > G	single base substitution	ETGE	R	ND	1
MU1817004	chr2:g.178098807T > G	single base substitution	ETGE	P	ND	1
MU112734927	chr2:g.178098809T > G	single base substitution	ETGE	A	ND	1
MU871364	chr2:g.178098803C > A	single base substitution	ETGE	V	ND	1
MU2689228	chr2:g.178098800T > G	single base substitution	ETGE	A	ND	1

**Table 2:** List of NRF2 target genes and their effects on HCC development.

NRF2 Target Genes	Effect of Gene Expression	ARE in Promoter
NAD(P)H dehydrogenase, quinone 1 (*NQO1*) and Hemeoxigenase 1 (*HO-1*) [92]	Constitutive activation of NRF2 by hepatotoxin contributes to the upregulation of *NQO1* and *HO-1*. This promotes liver cancer cell growth.	YES
B-cell lymphoma-extra-large (*Bcl-xL*) [68]	Antioxidant stabilized NRF2 increases the expression of *Bcl-xL* gene which causes reduction in apoptosis, increase cell survival, and drug resistance in Hepa1-6 cells.	YES
Glutathione S-transferase A4 (*GSTA4*) [93]. Glutamate-cysteine ligase (*GCLC*) [91], [94]	Constitutive activation of NRF2 in preneoplastic lesions of HCC increases the expression of *GSTA4* and *GCLC* and promotes HCC cell growth.	YES
Placental glutathione S-transferase (*GST-P*) [95,96]	NRF2/MafK heterodimer activates *GST-P* gene (a prominent tumor marker for hepatocarcinogenesis) through the binding with GPE1 enhancer during hepatocarcinogenesis.	YES
Matrix metalloproteinases-9 (*MMP-9*) [82], [97]	Up-regulation of NRF2 in HepG2 cells increases the *MMP-9* expression which promotes the cell invasion ability of HCC. In response to ROS, NRF2 induces *MMP-9* expression in HepG2 cells, which contributes to cancer cell migration and invasiveness.	YES
Platelet-Derived Growth Factor-A (*PDGFA*) [98]	NRF2 promotes *PDGFA* transcription by interacting with SP1 thus promotes HCC proliferation.	NO (Through interaction with Sp1)
Prostaglandin reductase-1 (*PTGR1*) [99]	*PTGR1* expression regulated by NRF2 regulates antioxidant responses to promote cell proliferation in HCC. PTGR1 overexpression in HCC increases cell proliferation and develop resistance to ROS-induced cell death.	YES
Peroxisome proliferator-activated receptor γ (*PPARγ*) [100]	Overexpression of NRF2 in HFD increases the expression of *PPARγ* and accumulates hepatic triglyceride which initiates NAFLD.	NO (Correlation was tested)
26S proteasome non-ATPase regulatory subunit 10 (*PSMD10*) or Gankyrin [101]	Increased NRF2 activity up-regulates gankyrin expression in HCC.	YES
Mouse double minute 2 homolog (*MDM2*) [71,102]	During HCV infection, NRF2 translocates into the nucleus and induces MDM2-mediated retinoblastoma protein (Rb) degradation. This induces HCC progression.	YES

## Abbreviations

AKR1B10	Aldo-Keto Reductase Family 1 Member B10
AKR1B15	Aldo-Keto Reductase Family 1 Member B15
ARE	Antioxidant response element
Atg5	Autophagy Related 5
Atg7	Autophagy Related 7
BAX	BCL2 Associated X
Bcl-xL	B-cell lymphoma-extra-large
Bcl2	B-cell lymphoma 2
bZIP	Basic leucine zipper
CRISPR/Cas9	Clustered regularly interspaced short palindromic repeats/ CRISPR-associated protein 9
CUL3 E3	Cullin 3-RING E3
DEN/CCl4	Diethylnitrosamine/Carbon tetrachloride
ERK	Extracellular signal-regulated kinases
FGF19	Fibroblast growth factor 19
FOXA1	Forkhead box protein A1
G6PD	Glucose-6-phosphate dehydrogenase
GCLC	Glutamate-Cysteine Ligase Catalytic Subunit
GCLM	Glutamate-Cysteine Ligase Modifier Subunit
GPX2	Glutathione Peroxidase 2
GSK3	Glycogen synthase kinase 3
GST-P	Placental glutathione S-transferase
GSTA4	Glutathione S-transferase A4
HBV	Hepatitis B virus
HCC	Hepatocellular carcinoma
HCV	Hepatitis C virus
HFD	High-fat diet
HO-1	Heme oxygenase-1
Huh-1	Human hepatoma cell line-1
ICGC	International Cancer Genome Consortium
JNK	c-Jun N-terminal kinases
KD	Knockdown
KEAP1	Kelch-like ECH-associated protein 1
KO	Knockout
LSCC	Lung Squamous Cell Carcinoma
Maf	Musculoaponeurotic fibrosarcoma
MAPK	Mitogen-activated protein kinase
Mdm2	Mouse double minute 2 homolog
MMP-9	Matrix metalloproteinase
MTHFD1L	Methylenetetrahydrofolate dehydrogenase 1–like
mTORC1	Mammalian target of rapamycin complex 1
NAFLD	Non-alcoholic fatty liver disease
NASH	Non-alcoholic steatohepatitis
NF-κB	Nuclear factor-kappa beta
NQO1	NAD(P)H-quinone oxidoreductase 1
NRF1	Nuclear respiratory factor 1
NRF2	NF-E2-related factor 2
NRF3	Nuclear factor-like factor 3
PDGFA	Platelet-Derived Growth Factor-A
PGD	Phosphogluconate Dehydrogenase
PI3K-Akt	Protein Kinase B Phosphoinositide-3-kinase
PPARα	Peroxisome proliferator-activated receptor alpha
PPARγ	Peroxisome proliferator-activated receptor γ
PPP	Pentose phosphate pathway
PRDX1	Peroxiredoxin 1
PSMD10	26S proteasome non-ATPase regulatory subunit 10
PTGR1	Prostaglandin reductase-1
Rb	Retinoblastoma protein
ROS	Reactive oxygen species
SQSTM1	Sequestosome-1
Smad3	SMAD family member 3
TALDO1	Transaldolase 1
TCGA	The Cancer Genome Atlas
TERT	Telomerase reverse transcriptase
TGF-β1	Transforming growth factor beta 1
TKT	Transketolase
TXNRD1	Thioredoxin Reductase 1
UHMK1	U2AF homology motif kinase 1
β-TrCP	Beta-transducin repeats-containing proteins
